# Cardiopatia Carcinoide: Relato de Caso e Revisão da Literatura

**DOI:** 10.36660/abc.20220245

**Published:** 2023-06-28

**Authors:** Isabela Bispo Santos da Silva Costa, Edielle de Sant`Anna Melo, Armando Furtado, Juliana Barbosa Sobral-Alves, Stephanie Itala Rizk, Luiz Alberto Benvenuti, Carlos E. Rochitte, Carlos Manuel de Almeida Brandão, Pablo Maria Pomarentzeff, Cristina Salvadori Bittar, Filomena Regina Barbosa Gomes Galas, José Otavio Costa Auler, Paulo Marcelo Gehm Hoff, Roberto Kalil, Fabio Biscegli Jatene, Ludhmila Abrahão Hajjar

**Affiliations:** 1 Universidade de São Paulo Instituto do Câncer do Estado de São Paulo São Paulo SP Brasil Universidade de São Paulo Instituto do Câncer do Estado de São Paulo, São Paulo, SP – Brasil; 2 Hospital das Clínicas Faculdade de Medicina Universidade de São Paulo São Paulo SP Brasil Instituto do Coração do Hospital das Clínicas da Faculdade de Medicina da Universidade de São Paulo, São Paulo, SP – Brasil; 3 Hospital Sírio-Libanês São Paulo SP Brasil Hospital Sírio-Libanês, São Paulo, SP – Brasil

**Keywords:** Síndrome do Carcinoide Maligno, Doença Cardíaca Carcinoide, Tumores Neuroendócrinos

## Introdução

A Síndrome Carcinoide (SC) é uma síndrome paraneoplásica frequentemente diagnosticada em pacientes com tumores neuroendócrinos (TNEs) associados com secreção de fatores humorais. Esses fatores incluem a serotonina (5-HT), a qual parece ser a substância mais comum associada à síndrome, além de histamina, calicreína, prostaglandinas e taquicininas.^1^ Entre os pacientes diagnosticados com TNEs localizados no duodeno e intestino delgado, cerca de 20% irão desenvolver SC durante o acompanahmento.^2^ A presença de SC está fortemente associada com maior grau e estágio do tumor, e associada com menor sobrevida em comparação a pacientes sem SC.^2^

Sinais e sintomas relacionados à SC incluem rubor, diarreia, dor abdominal, broncoespasmo, pelagra, cardiopatia carcinoide (CC) e fibrose mesentérica.^3^ A CC é caracterizada pelo envolvimento das válvulas cardíacas direitas (principalmente regurgitação tricúspide e pulmonar), levando à dilatação e disfunção do ventrículo direito (VD).^4^ Pacientes com uma alta carga de doença, como pacientes com tumores metastáticos geralmente apresentam cardiopatia. A presença de CC está associada a um pior prognóstico e a altas taxas de mortalidade.^5 , 6^ Os achados mais comuns da CC estão descritos na Figura Central .

**Figura Central:**
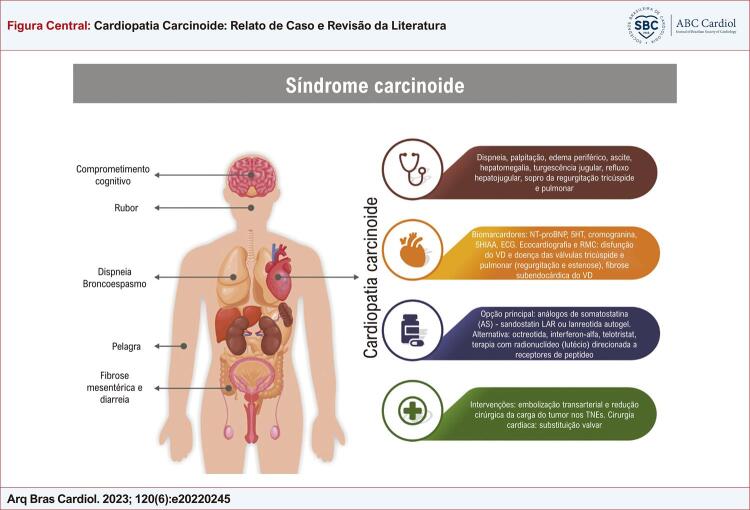
: Cardiopatia Carcinoide: Relato de Caso e Revisão da Literatura

Síndrome carcinoide (SC): as manifestações mais comuns da SC são comprometimento cognitivo, rubor, dispneia/broncoespasmo, pelagra, fibrose mesentérica, diarreia e cardiopatia carcinoide. No manejo da CC, é importante (a) reconhecer os sintomas, (b) realizar uma avaliação diagnóstica, (c) administrar terapia medicamentosa e (d) administrar terapia intervencionista; VD: ventrículo direito; NT-proBNP: fragmento N-terminal do peptídeo natriurético tipo B; 5HT: serotonina; 5HIAA: ácido 5-hidroxi-indolacético; AS: Análogos de Somatostatina; TNEs; Tumores Neuroendócrinos; ECG: eletrocardiograma.

O diagnóstico de CC é normalmente confirmado por exame de imagem cardíaca e biomarcadores. O manejo dos pacientes com CC é complexo. Enquanto oncologistas têm como objetivo controlar a doença sistêmica, reduzir a carga de tumor, e diminuir os níveis de neuro-hormônios, cardiologistas atuam na redução de sintomas e da carga da insuficiência cardíaca. Determinar o momento mais apropriado para a cirurgia cardíaca e a estratégia terapêutica perioperatória é essencial para melhorar os desfechos da doença. Nesta revisão, apresentamos o caso clínico de um paciente com CC e doença valvular grave. Desafios diagnósticos e a importância do reconhecimento e manejo precoce da doença carcinoide são pontos chaves na melhoria dos desfechos.

## Relato de Caso

Paciente de 21 anos do sexo masculino, sem história pessoal relevante apresentou insuficiência cardíaca em 2017, quando foi diagnosticado com estenose valvar pulmonar e regurgitação tricúspide moderada. O paciente submeteu-se à substituição da valva pulmonar por uma bioprótese em outra instituição em março de 2017. O paciente permaneceu assintomático por seis meses após o procedimento cirúrgico. Em seguida, o paciente desenvolveu dispneia progressiva e edema associado a rubor e diarreia. Em 2018, após investigação clínica, foi diagnosticado um tumor neuroendócrino no jejuno com meatástases hepáticas. A ecocardiografia transtorácica (ETT) mostrou regurgitação tricúspide grave com folhetos da valva tricúspide fixos e disfunção do Ventrículo Direito (VD). O paciente recebeu diagnóstico de SC, CC e insuficiência ventricular direita. O tratamento com injeções de acetato de lanreotida e diuréticos orais foi iniciado mensalmente, e o paciente apresentou melhora na classe funcional e controle da doença oncológica.

Contudo, em abril de 2021, o paciente foi admitido no Instituto do Coração (InCor) por insuficiência cardíaca aguda descompensada. O eletrocardiograma mostrou ritmo juncional de 44 bpm e sinais de sobrecarga do VD. Após tratamento com infusão de inotrópico (dobutamina), o paciente retornou ao ritmo sinusal. A radiografia de tórax revelou área cardíaca normal e congestão pulmonar. A concentração de peptídeo natriurético tipo-B (BNP) foi 338pg/mL (valor de referência < 100pg/mL), e a de ácido 5-hidroxi-indolacético (5-HIAA) foi 30,4mg/24 horas (valor de referência 2-6 mg/24 horas).

**Figura 1:**
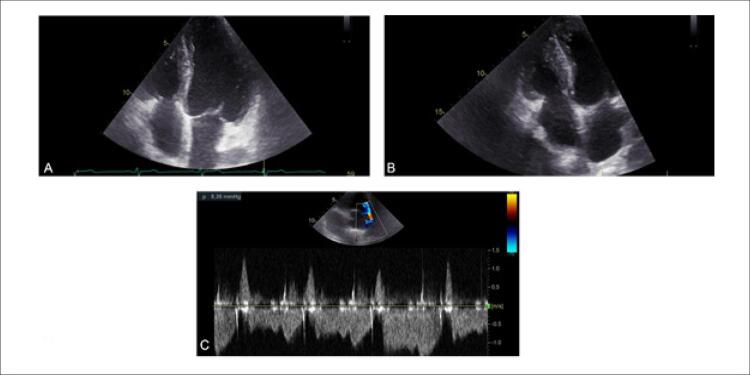
– Ecocardiografia transesofágica e transtorácica bidimensional. A) corte apical de quatro câmaras na sístole mostrando a válvula tricúspide aberta e retraída e a válvula mitral fechada; B) corte apical de quatro câmaras na sístole (foco no ventrículo direito) mostrando a válvula tricúspide aberta e retraída e a válvula mitral fechada; C) curva do fluxo tricúspide no Doppler contínuo, mostrando uma equalização da pressão entre o átrio direito e o ventrículo direito.

**Figura 2:**
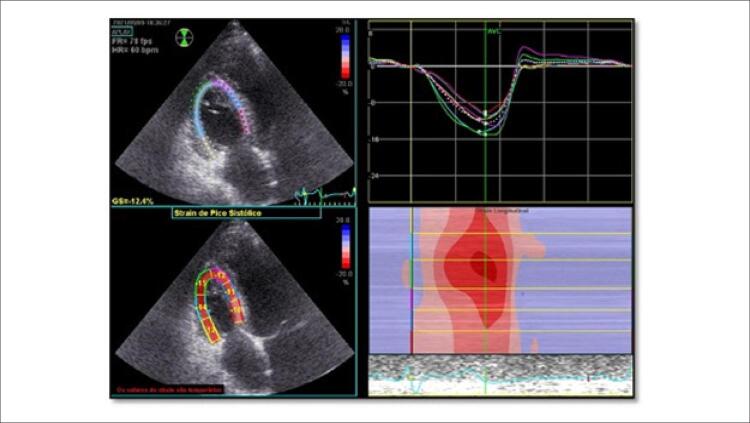
– Corte apical de quatro câmaras (foco no ventrículo direito); quantificação do strain ventricular direito por ecocardiografia speckle-tracking mostrando valores anormais do strain global e do strain da parede livre (12,4% e 13,6%, respectivamente).

O paciente submeteu-se à ETT bidimensional e ecocardiografia transesofágica, que mostraram câmara cardíaca e válvulas esquerdas normais ( Figura 1 ). As câmaras cardíacas direitas (diâmetro basal do VD: 41 mm; área do átrio direito: 19cm^2^) encontravam-se um pouco aumentadas, e se notava espessamento da parede livre do VD (9mm). A bioprótese valvular pulmonar apresentou função e gradientes normais. Os folhetos da válvula tricúspide encontravam-se espessados, imóveis e retraídas, resultando em uma válvula sem coaptação ( Figura 1 ). A variação da área do VD foi de 34%. Com o volume de amostra do Doppler de pulso posicionado no anel tricúspide lateral no corte apical de quatro câmaras focado no VD, obteve-se a velocidade de pico sistólico (VPS) por Doppler tecidual (9,5 cm/s). A excursão sistólica do plano anular tricúspide (TAPSE) foi registrada posicionando-se o cursor do modo M através da base do anel tricúspide lateral e quantificando seu movimento longitudinal, que foi de 16mm. Esses três parâmetros da função do VD encontravam-se no limite inferior de normalidade. Medidas do *strain* do VD (global e da parede livre) apresentaram valores normais de 12,4% e 13,6%, respectivamente ( Figura 2 ).

**Figura 3:**
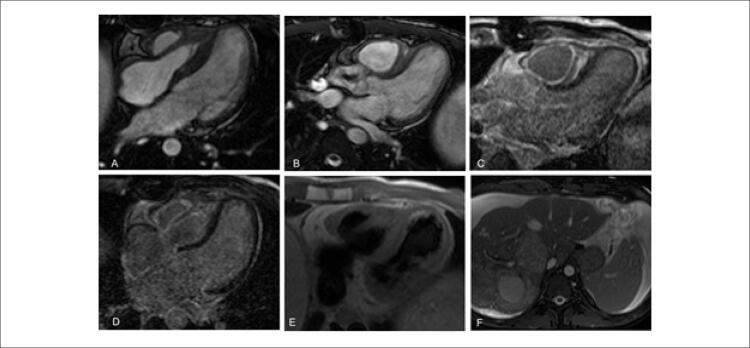
– Avaliação por ressonância magnética cardíaca; A e B) cine-ressonância magnética de quatro câmaras e três câmaras, respectivamente; há um aumento na espessura da parede livre do ventrículo direito e do músculo papilar e retração da válvula tricúspide. O átrio direito e o ventrículo direito aparecem como uma câmara única. O septo interventricular encontra-se deslocado para o ventrículo esquerdo, sugerindo um aumento do volume ventricular direito. C e D) mostram presença de realce tardio com gadolínio (RTG) na região subendocárdica da parede livre, músculo papilar e septo interventricular. Não se observou RTG no ventrículo esquerdo. E) técnica do sangue escuro (dark blood) mostrando aumento da espessura do miocárdio do ventrículo esquerdo e ausência de edema; F) imagens da transição toracoabdominal mostrando hepatomegalia, múltiplos nódulos hepáticos e ascite, característico de tumores neuroendócrinos.

A ressonância magnética cardíaca (RMC) identificou fração de ejeção ventricular direita normal (58%). A válvula tricúspide encontrava-se retraída e espessada, com mobilidade reduzida, resultando em regurgitação tricúspide grave. O átrio direito e o VD apareciam como uma cavidade única. As imagens adquiridas bem após a injeção de contraste mostraram realce tardio de gadolínio na parede atrial direita e padrões circunferenciais e difusos no VD, compatíveis com fibrose endocárdica ( Figura 3 ). Resultados da angiografia coronária por tomografia computadorizada foram normais.

Após melhora dos sintomas de insuficiência cardíaca, com o tratamento com diurético intravenoso e restrição de líquidos, o paciente submeteu-se à embolia arterial das lesões metastáticas hepáticas hipervascularizadas. O paciente recebeu alta com melhora clínica significativa. Apesar do controle da doença sistêmica, o paciente foi internado em setembro de 2021 por sintomas persistentes de insuficiência cardíaca para substituição cirúrgica da válvula tricúspide.

Uma equipe multidisciplinar composta de cardiologistas clínicos e cirurgiões, anestesiologistas e oncologista estabeleceu estratégias para reduzir a morbidade cirúrgica, incluindo medidas para prevenir uma crise carcinoide. Infusão intravenosa contínua de octreotida na dose de 100 µγ/h foi administrada 12 horas antes da cirurgia e mantida até 72 horas no pós-operatório. O manejo anestésico consistiu em terapia hemodinâmica avançada e anestesia intravenosa. Vasopressina e epinefrina foram usadas durante a cirurgia e por 48 horas após a cirurgia para controlar a síndrome vasoplégica e aumentar a frequência cardíaca e o inotropismo. Óxido nítrico inalatório foi usado para reduzir a resistência vascular pulmonar, reduzindo a pós-carga do VD. O paciente recebeu ainda complexo protrombínico (duas unidades) e albumina (100mL). Não foi necessária transfusão de sangue.

**Figura 4:**
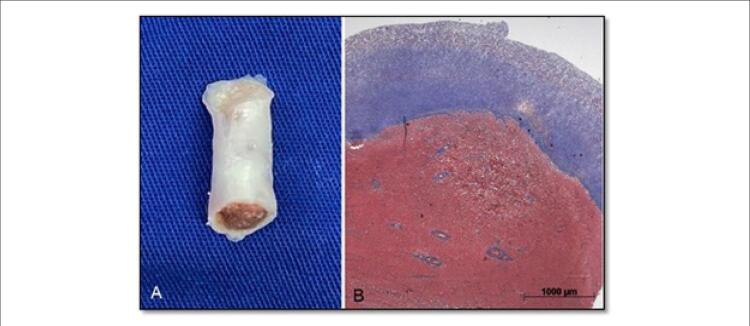
– Espécimes patológicos constituídos de um segmento cilíndrico do músculo papilar medindo 18mm, com presença de tecido esbranquiçado espesso em uma das extremidades (A). Seções histológicas transversais do músculo papilar revelaram um endocárdio fibroso espessado (B; corante tricromo de Masson).

Observou-se espessamento do pericárdio durante a cirurgia, e foi realizada pericardiectomia parcial. Os folhetos da válvula tricúspide não foram claramente identificados. Na topografia da válvula observou-se somente uma área extensa de atrofia e fibrose. Um segmento cilíndrico de músculo papilar foi ressecado e enviado para análise anatomopatológica, que revelou um endocárdio espesso e fibroso ( Figuras 4 e 5 ). A substituição da válvula tricúspide foi realizada usando uma bioprótese número 25.

**Figura 5:**
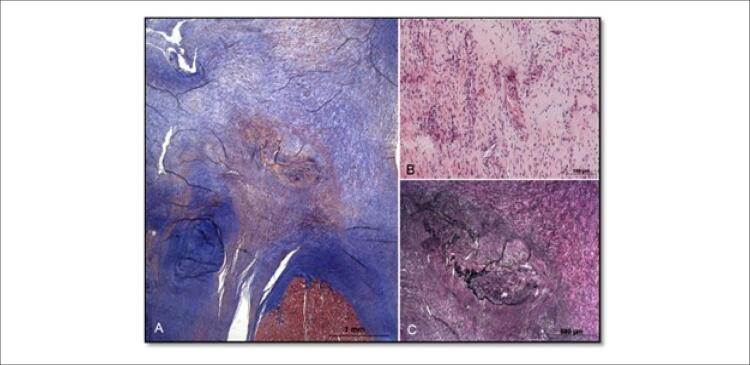
– Seções histológicas longitudinais do tecido esbranquiçado na ponta do músculo papilar mostra um tecido fibroso denso (A) corante tricromo de Masson), com um foco central de neovascularização, infiltração inflamatória crônica leve (B- coloração de hematoxilina-eosina) e algumas fibras elásticas irregulares (C- coloração de Verhoeff).

Após a cirurgia, o paciente desenvolveu choque vasoplégico com lesão renal aguda, tratada com sucesso com ressuscitação volêmica e drogas vasoativas sem necessidade de terapia renal substitutiva. Foram realizadas duas parecenteses durante a internação para o tratamento de ascite e hipertensão intra-abdominal. O paciente teve boa recuperação e recebeu alta sem sintomas, com função renal normal. O ETT mostrou função biventricular preservada e biopróteses tricúspide e pulmonar normais.

### Revisão da Literatura

TNEs são neoplasias raras, com uma incidência entre 2,5 a 5 casos por 1000000 habitantes. A maioria desses tumores são benignos, sendo que menos de 10% são malignos, e os pacientes com doença localizada apresentam prognóstico favorável, com uma taxa de sobrevida em cinco anos entre 78% e 93%.^7^ Eles ocorrem geralmente no trato gastrointestinal ou no sistema broncopulmonar.

Os TNEs gastrointestinais, originalmente denominados carcinoides, apresentam crescimento lento, causando pouco ou nenhum sintoma, até se tornarem grandes ou tenham criado metástases. O fígado é o local mais comum de metástase (cerca de 80%), e nos TNEs metastáticos, a sobrevida em cinco anos é baixa, variando entre 19% e 38%.^7^ Aproximadamente 30% a 40% dos pacientes apresentam características de SC, com episódios de alterações vasomotoras (rubor e hipotensão), diarreia, e broncoespasmo, devido à liberação de substâncias vasoativas, tais como serotonina, cininas, prostaglandinas, substância P e cromogranina A.^8^

A CC é uma complicação grave da SC, associada a altas taxas de mortalidade. Sua fisiopatologia envolve efeitos mitogênicos nos fibroblastos e células musculares lisas do coração, sob a ação de citocinas inflamatórias e o aumento do fator decrescimento transformador-β_1_ induzido por 5-HT via ativação dos receptores 5-HT.^9^ Um diagnóstico precoce do envolvimento cardíaco, uma estratégia multimodal de cuidado perioperatório, e uma substituição em tempo hábil da válvula cardíaca são essenciais para melhorar o prognóstico dos pacientes com TNEs e envolvimento cardíaco.

Níveis elevados de 5-HIAA, BNP e cromogranina A são úteis biomarcadores na avaliação do envolvimento cardíaco e progressão da doença.^10 - 13^ Embora a cromogranina A não seja um bom marcador para o diagnóstico, é útil na avaliação de acompanhamento para ocorrência de neoplasias recorrentes.^14 - 16^ Em pacientes com SC, o fragmento N-terminal do peptídeo natriurético tipo B (NT-proBNP) é um marcador específico e sensível para a presença de CC na ausência de outras doenças cardíacas.^17^ Recomenda-se que todos os pacientes com SC seja submetido à quantificação de NT-proBNP a cada seis a 12 meses para detectar sinais precoces de CC.^17 , 18^ Ainda, o 5-HIIA é o produto final do metabolismo da serotonina, e pode ser medido pelo exame de urina 24 horas. Trata-se de um teste diagnóstico inicial útil para SC, principalmente para identificar pacientes em risco para desenvolver CC.^19^ A medida 5-HIIA plasmático ou na urina de 24 horas é mandatória para o diagnóstico e seguimento de SC.

A ecocardiografia é o exame de imagem de escolha no diagnóstico e no acompanhamento da CC. O exame pode ser usado para caracterizar doença da válvula tricúspide e estimar o tamanho e função do VD e o tamanho do átrio direito. O envolvimento típico da válvula tricúspide é caracterizado pela presença de um anel dilatado e folhetos difusamente espessados e retraídos, que não fecham durante a sístole, sem completa abertura na diástole, exibindo mobilidade limitada. Aproximadamente 80% dos pacientes apresentam regurgitação ou estenose da válvula pulmonar, com cúspides difusamente espessadas, e vários graus de retração e reduções na excursão. A análise do *strain* e a ecocardiografia são técnicas complementares para confirmar a gravidade das lesões valvares e avaliar o prognóstico.

A RMC é o método padrão-ouro para a quantificação da função biventricular. A avaliação da fração de ejeção do VD por RMC é essencial na tomada de decisões sobre a cirurgia. A partir da RMC em pacientes com CC, é possível (a) melhorar a caracterização das válvulas, (b) avaliar os volumes regurgitantes, os tamanhos das câmaras e a função do VD, (c) identificar e quantificar a fibrose, (d) diagnosticar metástase no miocárdio, e (e) auxiliar na definição do tratamento cirúrgico.^20^ Os achados típicos na RMC são espessamento da válvula tricúspide e/ou dos folhetos da válvula pulmonar com defeitos de coaptação.^21^ Utilizando contraste de fase, é possível estimar os volumes de regurgitação e classificar o envolvimento da válvula como leve, moderado ou grave.^21^ Neste paciente, a RMC mostrou um envolvimento difuso raro do VD, com fibrose na válvula tricúspide, miocárdio e no músculo papilar.

A Tomografia Computadorizada (TC) cardíaca pode ser realizada em pacientes com CC como um método complementar. É especialmente indicada quando há envolvimento da válvula pulmonar, e permite a avaliação de doença arterial coronariana. Ainda, a TC cardíaca pode diagnosticar metástases e analisar sua relação com o coração e os vasos, e identificar complicações como pericardite constritiva.^22^

O manejo dos pacientes com CC inclui dois pontos principais – o controle de TNEs e o tratamento da insuficiência cardíaca. Para controle do sintoma, reduzir os níveis hormonais ou a carga do tumor é essencial, e o tratamento sistêmico com análogos de somatostatina (AS) de longa duração é indicado. Hofland et al.,^23^ em uma meta-análise recente, mostraram que os AS, tais como octreotida e lanreotida, levaram à melhora dos sintomas em 65-72% dos pacientes, e à resposta bioquímica em 45-46% dos pacientes. Um ajuste na dose ou na frequência, ou a troca entre classes levou à redução dos rubores e/ou diarreia em 72-85% dos casos.^23^

Na doença refratária, a terapia local deve ser considerada para pacientes com doença hepática dominante, com possibilidade de citorredução. Nesses casos, é possível intervenção com ressecção, ablação por radiofrequência, embolização branda, quimioembolização ou radioembolização. Em pacientes a quem a terapia direcionada é contraindicada, terapias alternativas com doses progressivas de AS, aumentando-se a frequência de injeção ou alterando-se o AS. Para pacientes com diarreia frequente apesar do uso de medicamentos antidiarreicos, o telotristat etil é recomendado. Em casos raros, em pacientes com sintomas refratários, a terapia com radionuclídeos do receptor de peptídeo e interferon-alfa pode ser aplicada.^23^

Em pacientes com doença valvar grave, identificada por ecocardiografia ou RMC, deve-se considerar o tratamento cirúrgico. Em pacientes com regurgitação tricúspide e pulmonar grave, a deterioração progressiva da função do VD e sintomas persistentes de insuficiência cardíaca causada por CC favorecem o tratamento cirúrgico. É essencial consultar o oncologista no momento da decisão terapêutica para avaliar o controle adequado da SC e a expectativa de vida para ponderar os riscos e os benefícios da cirurgia.^21^

O cuidado perioperatório é essencial para prevenir crise carcinoide e complicações. A crise carcinoide é uma manifestação letal da SC caracterizada por profunda instabilidade autonômica em situações de liberação de catecolaminas causada por estresse, manipulação do tumor, ou anestesia. Nesse cenário, pode ocorrer choque cardiogênico por disfunção ventricular direita, com altas taxas de mortalidade.^24^

Outras complicações pós-operatórias associadas são hipertensão pulmonar, choque vasoplégico, sangramento e insuficiência renal aguda. Recomenda-se o uso de octreotida intravenosa durante o período perioperatório.^25^ A medicação também é importante para prevenir hipercapnia, hipotermia, hipoxemia e instabilidade hemodinâmica. Nesses pacientes, sugerem-se monitores de débito cardíaco para avaliar o status hídrico, otimizar o índice cardíaco e prevenir hipóxia tecidual. No choque vasoplégico, hidrocortisona (100mg a cada oito horas) e vasopressina intravenosa (0,04 U – 0,06 u/hora) pode ser consideradas.

Este caso ilustra o importante papel da suspeita clínica no diagnóstico correto da CC. Sintomas e sinais de insuficiência do VD, biomarcadores cardíacos elevados, e anormalidades na válvula tricúspide e pulmonar, avaliados por ecocardiografia e RMC são sugestivos de CC. É necessária interação contínua entre cardiologistas, oncologistas, e cirurgiões para melhor controle da CC e para o estabelecimento das melhoras terapias por meio de uma avaliação personalizadas dos pacientes.

## Conclusão

A CC é uma doença potencialmente grave que envolve principalmente válvulas cardíacas direitas, e leva à insuficiência cardíaca e piores desfechos em pacientes com TNEs. Técnicas mais avançadas de imagem cardiovascular, tais com ecocardiografia *speckle tracking* e RMC melhoraram a acurácia diagnóstica e a detecção precoce de disfunção cardíaca. Uma equipe envolvendo cardiologista, oncologista, cirurgiões, enfermeiros e anestesiologistas é essencial para o manejo de CC para alcançar controle de sintomas e maiores taxas de sobrevida e qualidade de vida.
