# Supplementary material for: Cardiopatia Carcinoide: Relato de Caso e Revisão da Literatura
Source: Arq Bras Cardiol. 2023 Jun 28;120(6):e20220245. [Article in Portuguese] doi: 10.36660/abc.20220245 (PMC10365021; doi:10.36660/abc.20220245)
Supplement: Translation

# Translation: Carcinoid Heart Disease: A Case Report and Literature Review

**Authors:** Isabela Bispo Santos da Silva Costa, Edielle de Sant`Anna Melo, Armando Furtado, Juliana Barbosa Sobral-Alves, Stephanie Itala Rizk, Luiz Alberto Benvenuti, Carlos E. Rochitte, Carlos Manuel de Almeida Brandão, Pablo Maria Pomarentzeff, Cristina Salvadori Bittar, Filomena Regina Barbosa Gomes Galas, José Otavio Costa Auler, Paulo Marcelo Gehm Hoff, Roberto Kalil, Fabio Biscegli Jatene, Ludhmila Abrahão Hajjar

**Affiliations:** 1 Brazil Universidade de São Paulo Instituto do Câncer do Estado de São Paulo, São Paulo, SP – Brazil; 2 Brazil Instituto do Coração do Hospital das Clínicas da Faculdade de Medicina da Universidade de São Paulo, São Paulo, SP – Brazil; 3 Brazil Hospital Sírio-Libanês, São Paulo, SP – Brazil

**Keywords:** Malignant Carcinoid Syndrome, Carcinoid Heart Disease, Neuroendocrine Tumors

## Introduction

Carcinoid syndrome (CS) is a paraneoplastic syndrome frequently diagnosed in patients with neuroendocrine tumors (NETs) associated with the secretion of humoral factors. These factors include serotonin (5-HT), which appears to be the most common substance associated with the syndrome, as well as histamine, kallikrein, prostaglandins, and tachykinins.^1^ Among patients diagnosed with NETs located in the duodenum and small intestine, approximately 20% will develop CS during follow-up.^2^ The presence of CS is strongly associated with a higher tumor grade and stage and is associated with shorter survival than in patients without CS.^2^

Symptoms and signs related to CS are flushing, diarrhea, abdominal pain, bronchospasm, pellagra, carcinoid heart disease (CHD) and mesenteric fibrosis.^3^ CHD is characterized by the involvement of the right-sided heart valves (mostly tricuspid and pulmonary regurgitation), leading to right ventricular (RV) dilatation and dysfunction.^4^ Heart disease is usually found in patients with a high burden of disease, notably patients with metastatic tumors. The presence of CHD is associated with a worse prognosis and high mortality rates.^5 , 6^ The most common findings of CS are described in the Central Illustration .

**Central Illustration:** : Carcinoid Heart Disease: A Case Report and Literature Review

Carcinoid Syndrome: The most common manifestations present in CS are cognitive impairment, flushing, dyspnea/bronchospasm, pellagra, mesenteric fibrosis, diarrhea and carcinoid heart disease. In the management of CHD, it is important to (a) recognize the symptoms, (b) perform a diagnostic evaluation, (c) administer drug treatment and (d) administer interventional treatment. RV: right ventricular; NT-proBNP: N-terminal pro-brain-type natriuretic peptide; 5HT: serotonin; 5HIAA: 5-hydroxyindoleacetic acid; SSAs: somatostatin analogues; NETs: neuroendocrine tumors

The diagnosis of CHD is usually confirmed by cardiac imaging and biomarkers. The management of patients with CHD is complex. Oncologists aim to control the systemic disease, reduce the tumor load, and decrease the neurohormone levels, while cardiologists act to reduce the symptoms and the burden of heart failure. Determining the appropriate time for cardiac surgery and the optimal perioperative care is essential to improve disease outcomes. In this review, we report a clinical case of a CHD patient with severe valvular disease. Diagnostic challenges and the importance of the prompt recognition and management of carcinoid disease are key points to improve outcomes.

## Case Report

A 21-year-old man with no relevant personal history presented with heart failure in 2017, when he was diagnosed with severe pulmonary valve stenosis and moderate tricuspid regurgitation. He underwent pulmonary valve replacement with a bioprosthesis at another institution in March 2017. The patient remained asymptomatic for six months after the surgical procedure. Then, he developed progressive dyspnea and edema associated with flushing and diarrhea. In 2018, after clinical investigation, a neuroendocrine tumor of the jejunum with hepatic metastases was diagnosed. The transthoracic echocardiogram (TTE) showed severe tricuspid regurgitation with fixed tricuspid leaflets and right ventricular (RV) dysfunction. The patient had a diagnosis of CS and CHD, with RV heart failure. Treatment with lanreotide injections and oral diuretics was started monthly, and the patient presented functional class improvement and oncologic disease control.

However, in April 2021, the patient was admitted to the InCor due to acute decompensated heart failure. The electrocardiogram showed a junctional rhythm of 44 bpm and signs of RV overload. After treatment with inotropic infusion (dobutamine), the patient returned to sinus rhythm. Chest X-ray revealed a normal cardiac area and pulmonary congestion. The B-type natriuretic peptide (BNP) level was 338 pg/mL (reference < 100 pg/mL), and 5-hydroxyindoleacetic acid (5-HIAA) was 30.4 mg/24 h (reference 2–6 mg/24 h).

The patient underwent two-dimensional (2D) TTE and transesophageal echocardiogram, which showed a normal left cardiac chamber and valves ( Figure 1 ). The right cardiac chambers (basal diameter of the right ventricle: 41 mm; right atrium area: 19 cm^2^) were mildly enlarged, and the RV free wall thickening was noticeable (9 mm). The pulmonary bioprosthesis had a normal function and gradients. The leaflets of the tricuspid valve were thickened, immobile and retracted, leading to a valve without coaptation ( Figure 1 ). The RV fractional area change (FAC) was 34%. With the pulsed-wave Doppler sample volume positioned at the lateral tricuspid annulus in the RV focused apical four-chamber view, the peak systolic velocity (PSV) by tissue Doppler was obtained (9.5 cm/s). The tricuspid annular plane systolic excursion (TAPSE) was recorded by placing the M-mode cursor through the base of the lateral tricuspid annulus, and measuring its longitudinal motion at peak systole, which was 16 mm. These three parameters of RV function were at the lower limit of normality. The RV strain measurements (global and free wall strain) showed abnormal values of 12.4% and 13.6%, respectively ( Figure 2 ).

**Figure 1:** – Two-dimensional transthoracic and transesophageal echocardiographic evaluation; A) apical four-chamber view in systole displaying an opened and retracted tricuspid valve, while the mitral valve is closed; B) apical four-chamber view in systole (focused on the right ventricle) displaying an opened and retracted tricuspid valve, while the mitral valve is closed; C) triscupid flow curve, by continuous Doppler, showing a pressure equalization between the right atrium and ventricle.

**Figure 2:** – Apical four-chamber view (focused on the right ventricle); quantification of right ventricular strain by speckle-tracking echocardiography showing abnormal global and free wall strain values (12.4% and 13.6%, respectively).

Cardiac magnetic resonance imaging (CMRi) identified a normal RV ejection fraction (58%). The tricuspid valve was retracted and thickened, with reduced mobility, resulting in severe tricuspid regurgitation. The right atrium and the right ventricle appeared as a single cavity. The images acquired late after contrast injection showed the presence of late gadolinium enhancement (LGE) imaging in the right atrial wall and circumferential and diffuse patterns in the right ventricle, compatible with endocardial fibrosis ( Figure 3 ). The coronary tomography angiogram was normal.

**Figure 3:** – Cardiac magnetic resonance imaging evaluation. A and B) cine resonance magnetic imaging in 4-chamber and 3-chamber, respectively; there is an increase in the thickness of the free wall of the right ventricle and papillary muscle and a retracted tricuspid valve. The right atrium and the right ventricle appear as a single chamber. The interventricular septum is displaced to the left ventricle, suggesting an increased volume of the right ventricle. C and D) show the presence of late gadolinium enhancement (LGE) in the subendocardial region of the free wall, papillary muscle and interventricular septum. There was no LGE in the left ventricle. E) dark blood sequence with increased thickness of the right ventricular myocardium and absence of edema. F) images of the thoracoabdominal transition showing hepatomegaly, multiple hepatic nodules and ascites, characteristic of neuroendocrine tumors.

After improvement of the heart failure symptoms with intravenous diuretic treatment and fluid restriction, the patient underwent arterial embolization of the hypervascular liver metastatic lesions. He was discharged with significant clinical improvement. Despite control of systemic disease, due to persistence of symptoms of heart failure, in September 2021, the patient was admitted for surgical replacement of the tricuspid valve.

A multidisciplinary team composed of cardiologists, cardiac surgeons, anaesthesiologists and oncologists established strategies to reduce surgical morbidity, including measures to prevent a carcinoid crisis. Continuous intravenous octreotide at a dose of 100 µγ/h was administered 12 hours before surgery and was maintained until 72 hours postoperatively. Anesthetic management consisted of advanced hemodynamic therapy and intravenous anesthesia. Vasopressin and epinephrine were used during surgery and for 48 hours postoperatively to control the vasoplegic syndrome and to increase the heart rate and inotropism. Inhaled nitric oxide was used to decrease the pulmonary vascular resistance, reducing the RV afterload. Intraoperatively, the patient also received prothrombin complex (two units) and albumin (100 mL). No blood transfusion was needed.

A thickened pericardium was observed during the surgery, and a partial pericardiectomy was performed. The tricuspid valve leaflets were not clearly identified. In the valve topography, there was only an extensive area of atrophy and fibrosis. A cylindrical segment of papillary muscle was resected and sent for anatomopathological analysis, which revealed a thickened and fibrous endocardium ( Figures 4 and 5 ). Tricuspid valve replacement was performed using bioprosthesis number 25.

**Figure 4:** – The pathological specimen was constituted by a cylindrical segment of the papillary muscle measuring 18 mm, presenting a thickened, whitish tissue at one end (A). Transversal histological sections of the papillary muscle revealed a thickened, fibrous endocardium (B; Masson trichrome stain).

**Figure 5:** – Longitudinal histological sections of the whitish tissue at the tip of the papillary muscle show a dense fibrous tissue (A- Masson trichrome stain), with a central focus of neovascularization, mild chronic inflammatory infiltration (B- hematoxylin-eosin stain) and few irregular elastic fibers (C- Verhoeff stain).

After surgery, the patient developed vasoplegic shock with acute kidney injury, which was managed successfully with fluid resuscitation and vasoactive drugs without the need for renal replacement therapy. Two paracenteses were performed during the hospital stay to manage ascites and intraabdominal hypertension. The patient recovered well and was discharged without symptoms, with normal renal function. The TTE showed preserved biventricular function and normal tricuspid and pulmonary bioprostheses.

### Literature Review

Neuroendocrine tumors are rare neoplasms, with an incidence ranging from 2.5 to 5 cases per 100,000 population. Most such tumors are benign, with less than 10% being malignant, and for patients with localized disease, the prognosis is favorable, with a 5-year survival ranging from 78% to 93%.^7^ They typically arise from the gastrointestinal tract or the bronchopulmonary system.

Gastrointestinal NETs were originally named “carcinoids”; they usually grow slowly and cause few or no symptoms until they are large or have metastasized. The liver is the most common site of metastasis (approximately 80%), and in metastatic NETs, 5-year survival is poor, ranging from 19% to 38%.^7^ Approximately 30% to 40% of patients present with features of CS, with episodes of vasomotor alterations (flushing and hypotension), diarrhoea, and bronchospasm due to the release of vasoactive substances, such as serotonin, kinins, prostaglandins, substance P and chromogranin A.^8^

CHD is a severe complication of CS, associated with high rates of mortality. The pathophysiology involves mitogenic effects on fibroblasts and the cardiac smooth muscle cells, under the action of inflammatory cytokines and the upregulation of transforming growth factor-β_1_induced by 5-HT through activation of 5-HT receptors.^9^ Early diagnosis of cardiac involvement, a multimodal strategy of perioperative care and timely heart valve replacement are essential to improve the prognosis of NETs patients with cardiac involvement.

Biomarkers such as increased urinary 5-HIAA levels, BNP and chromogranin A are useful for evaluating cardiac involvement and disease progression.^10 - 13^ Chromogranin A is not a good marker for diagnosis, but it is useful for follow-up evaluation for recurrent neoplasms.^14 - 16^ In patients with CS, N-terminal pro-brain natriuretic peptide (NT-proBNP) is a sensitive and specific marker for the presence of CHD in the absence of other cardiac diseases.^17^ It is recommended that all patients with CS undergo 6- to 12-month evaluations of serum NT-proBNP to detect early signs of CHD.^17 , 18^ In addition, 5-HIAA is the final product of serotonin metabolism, and can be measured by 24-h urinary excretion. It is a useful initial diagnostic test for CS, especially to identify patients who are at risk for the development of CHD.^19^ The measurement of either 24-h urine 5-HIAA or plasma 5-HIAA is mandatory for the diagnosis and follow-up of CS.

Echocardiography is the imaging modality of choice for CHD diagnosis and follow-up. The test can be used to characterize tricuspid valve disease and to estimate the RV size and function and the size of the right atrium. The typical tricuspid valve involvement is characterized by a dilated annulus and diffusely thickened and retracted leaflets, which do not close during systole, without complete opening in diastole, exhibiting limited mobility. Approximately 80% of patients have pulmonary valve regurgitation or stenosis, with valve cusps diffusely thickened, and varying degrees of retraction and excursion reduction. Strain analysis and oesophageal echocardiography are complementary techniques to confirm the severity of the valve lesions and to assess the prognosis.

CMR is the gold standard method for quantifying biventricular function. The evaluation of the right ventricular ejection fraction by CMR is essential for making decisions about surgery. By performing CMR in patients with CHD, it is possible to (a) improve the characterization of the valves, (b) assess the regurgitant volumes, chamber sizes and RV function, (c) identify and quantify the fibrosis, (d) diagnose the myocardial metastasis and (e) assist in defining the surgical treatment.^20^ The typical findings in CMRi are thickened tricuspid and/or pulmonary valve leaflets with coaptation defects.^21^ Using phase contrast imaging, it is possible to estimate the regurgitation volumes and to classify the valve involvement as mild, moderate, or severe.^21^ In this patient, the CMRi showed rare diffuse RV involvement, with fibrosis in the tricuspid valve, myocardium, and papillary muscle.

Cardiac computed tomography (CT) can be performed in patients with CHD as a complementary method. It is especially indicated for involvement of the pulmonary valve, and it allows for the evaluation of coronary artery disease. Additionally, cardiac CT can diagnose metastases and analyze their relationship with the heart and vessels and may identify complications such as constrictive pericarditis.^22^

The management of patients with CHD includes two main aspects: the control of NETs and the treatment of heart failure. For symptom control, reducing the hormone levels or the tumor load is essential, and systemic treatment with long-acting somatostatin analogues (SSAs) is indicated. Hofland et al.,^23^ in a recent meta-analysis, showed that SSAs, such as octreotide and lanreotide, resulted in symptomatic improvement in 65-72% and in a biochemical response in 45-46% of patients. An adjusted dose or frequency or interclass switch led to a reduction in flushes and/or diarrhea in 72-85% of cases.^23^

In refractory disease, local therapy should be considered for patients with liver-dominant disease with the possibility of cytoreduction. In these cases, interventional treatment with resection, radiofrequency ablation, bland embolization, chemoembolization or radioembolization is possible.^23^ In patients for whom liver-directed therapy is not indicated, alternative treatments are escalating SSA doses, increasing the injection frequency or switching the SSA. For patients with frequent diarrhea despite antidiarrheals, teloristat ethyl is recommended. In rare cases, in patients with refractory symptoms, peptide receptor radionuclide therapy and interferon-alpha can be applied.^23^

In patients with severe valve disease by echocardiography or CMRi, surgery must be considered. In patients with severe tricuspid and pulmonary regurgitation, progressive deterioration of RV function and persistent heart failure symptoms caused by CHD favor surgical therapy. It is essential to consult the oncologist at the time of the therapeutic decision to assess the adequate control of the CS and the life expectancy to balance the risks and benefits of surgery.^21^

Perioperative care is essential to avoid a carcinoid crisis and complications. Carcinoid crisis is a life-threatening manifestation of CS characterized by profound autonomic instability in the setting of catecholamine release due to stress, tumor manipulation, or anesthesia. In this scenario, cardiogenic shock due to RV dysfunction might occur, resulting in high rates of mortality.^24^

Other associated postoperative complications are pulmonary hypertension, vasoplegic shock, bleeding and acute renal failure. It is recommended to use intravenous octreotide during the perioperative period.^25^ It is also important to avoid hypercapnia, hypothermia, hypoxemia and hemodynamic instability. In these patients, cardiac output monitors are suggested to assess the fluid status, to optimize the cardiac index and to prevent tissue hypoxia. In vasoplegic shock, hydrocortisone (100 mg every 8 hours) and intravenous vasopressin (0.04 U – 0.06 U/h) might be considered.^25^

This case illustrates the important role of clinical suspicion in correctly diagnosing CHD. RV heart failure symptoms and signs, elevated cardiac biomarkers, and abnormal tricuspid and pulmonary valves assessed by echocardiography and CMR suggest the diagnosis of CHD. Continuous interaction between cardiologists, oncologists and surgeons is required to better control CHD and to establish the best therapies through a personalized evaluation of the patients.

## Conclusion

CHD is a potentially severe disease involving mainly right-sided valves, leading to heart failure and worse outcomes in patients with NETs. Advanced cardiovascular imaging, such as speckle-tracking echocardiography and CMRi, has improved diagnostic accuracy and early detection of cardiac dysfunction. A heart team involving cardiologist, oncologist, surgeons, nursers, and anesthetist is essential for the management of CHD to achieve symptom control and increased survival rates and quality of life.
